# Progress in Medicine

**Published:** 1896-07-18

**Authors:** 


### Progress in Medicine.

### DISEASES OF THE CIRCULATORY SYSTEM.

**Pericardial Effusion.**—The diagnostic signs of large pericardial effusions have been studied by Ewart,[1] and his observations should prove valuable in cases where surgical interference is contemplated. Pericardial distension taking place usually in all directions, vertically as well as transversely, the first requisite is knowledge of the normal levels and lateral extensions of the præcordial dulness. The author gives the following signs: (1) Considerable extension of the lateral boundaries of the total area of dulness. (2) Great extension of the absolute dulness, the sternum absolutely dull. (3) Depression of the liver. (4) Dr. Rotch's sign—dulness in the right fifth intercartilaginous space; but Ewart cites a case which shows that Rotch's sign is not always to be depended upon, and therefore could not, as had been contended, be upheld as pathognomonic. (5) Differential diagnosis between pericardial effusion and cardiac dilatation, viz., the lower angle of the pericardial dulness projects towards the right. (6) The relation of the heart's apex to the left lower angle of dulness. In cases of cardiac enlargement or displacement to the left, however brought about, the apex beats at the extreme left limit of the dulness and at its lowest level. This is not the case in pericardial effusion, for then the apex cannot be felt where there is much effusion, but may be heard beating at a spot somewhat inside and above the boundaries of dulness. Ewart warns us against what he characterises as a remarkable misconception hitherto perpetuated by the text-books as to the alleged elevation of the apex within the pericardial effusion, even as high as the third interspace. This impulse, however, is not that of the apex of the heart, but rather of its base. Ewart maintains that at the usual spot for the apex in normal conditions it will also be found at the necropsy of any uncomplicated case of pericardial effusion. The impossibility of the apex being raised to the third interspace by the operation of gravitation or of ordinary mechanics is obvious, firstly, because the specific gravity of the heart, either filled with blood or empty, is much more than 1,018, which, according to Halliburton, is the usual density of pericardial effusions; secondly, we must remember that the heart is tethered to the bottom of the pericardium by the attachment of the inferior vena cava to the foramen quadratum in the central tendon, and that a considerable descent of the diaphragm must depress the level of the right auricle and tend to depress the apex far from allowing it to rise. (7) The first rib sign: In all cases of considerable pericardial effusion which Ewart has examined for this sign, it was possible to feel with the finger the upper edge of the first rib as far as its sternal attachment. (8) Posterior patch of pericardial dulness, a patch of marked dulness at the left inner base, extending from the spine for varying distances outwards, usually not quite so far as the scapular line and ceasing abruptly with a vertical outer boundary. Its shape is that of a square, and it is quite unlike that of any dulness arising from pleuritic effusion. (9) Tubular breathing below the right mamma: Although not constant, this sign does not appear to have been noticed, and Ewart states that it should be looked for in severe cases. (10) Posterior patch of tubular breathing and ægophony immediately below or slightly to the left of the tip of the left scapula. This sign also occurs in pleural effusion. (11) Secondary pleural effusions. (12) Large and slapping pulse of pericardial effusion. In his concluding remarks the author stated that the most difficult and most anxious part of the subject was the fact that in practice we have always to be prepared to find a wide difference between the classical account of disease and the individual case. Thus effusion may supervene either a previous attack which has left the heart adherent to part of the sac, or after an early fibrinous stage, more than usually agglutinative soft adhesion and partial filling of the sac with heavy gelatinous masses of fibrin. To such cases the ordinary rules as to the position of the heart do not apply.

**Dropsy.**—The factors in the production of dropsy, till recently considered so simple, have been the subject of many careful investigations, and in the Arris and

Gale lectures by Starling,[2] the present state of knowledge of this symptom has been ably summarised. Starling first reminds us that in health the two processes of lymph production by transudation from the blood plasma circulating in the capillaries, and of lymph absorption by the lymphatics, are exactly proportional, and that dropsy depends on a loss of balance between these two processes. Ludwig endeavoured to prove that the amount of lymph produced in any given part must be proportional to the difference between the pressure in the capillaries and the pressure in the extra-vascular spaces. On leading defibrinated blood through a limb, the lymph production in the limb was found proportional to the pressure at which the blood was led through it. After critically reviewing the work of Heidenhain and others, Starling points out that from this study of the conditions of lymph-production in the various parts of the body we must conclude that the endothelial cells of the vessels take no active part in the production, their vital activities being confined to the maintenance of their integrity as a filtering membrane with properties differing according to the part of the body in which they happen to be situated. The amount and composition of the lymph transuded in any part are determined solely by two factors: (1) the permeability of the vessel wall, and (2) the intracapillary blood pressure. The more permeable the capillary the greater is the amount of lymph transuded under any given pressure, the greater is its concentration in proteids, and the more easily is the amount of lymph altered by slight changes of pressure.

Passing on to the second physiological factor, the absorption of lymph, Starling reminds us that two ways of absorption have been described: (1) by the lymphatics; (2) by the blood vessels; and there can be no doubt that absorption may take place by both these ways. During normal existence the flow of lymph along the lymph channels, and thus the emptying of the deeper lymph channels, preparing them to receive fresh supplies from the tissues, depends on muscular movement. The system of valves in the lymph ducts prevents the return of lymph towards the empty lymph channels in relaxation following the muscular contractions which by compression emptied the channels in the first place. It is, in fact, impossible without such aid as muscular movements or passive flexion and extension of the limb to get any lymph-flow at all from a cannula placed in the lymphatics of the leg or arm.

As regarding the absorption of lymph by the blood vessels, Starling cites Majendie's experiments, which have been continued and extended by Ascher, Tubby, and himself. Tubby and Starling found, for example, that after injecting methylene blue or indigo carmine into the pleura the dye stuff appeared in the urine within five minutes, whereas the lymph presented no trace of blue for another twenty minutes or even two hours. It is evident, then, that in this case the dye must have been taken up by the blood vessels, and not by the lymphatics, and that this vascular absorption takes place with extreme rapidity. These facts, together with a later series of experiments by Leathes, which showed that after introduction of various salt solutions into the serous cavities, an interchange of constituents takes place directly between the blood and the injected fluids till the latter very shortly becomes isotonic with the blood plasma, are used to illustrate a fact of great interest and importance. In this mode of absorption by the blood vessels the so-called absorption really consists in an interchange by simple processes of diffusion, between blood and extravascular fluids. This power of exchange is probably of great importance in the normal metabolism of tissue. Starling cites as an instance the hypothesis that a cell is storing up in itself a large amount of calcium or secreting it away from the tissue fluids into the lumen of a gland duct, it is evident that the percentage amount or partial tension of the calcium salts in the fluid surrounding the cell wall will be diminished; then diffusion currents will be set up, and there will be a flow of calcium from the blood to the lymph, until the normal calcium tension is restored. We must conclude, therefore, that for the ordinary metabolic activities of the tissues no lymph flow at all is necessary, the cell being able to maintain all its diffusible food stuffs from the blood by means of diffusion, even through a stationary layer of lymph.

But in the case of dropsical fluids the exudation resembles in all points blood plasma, the only difference between the two consisting in the fact that the dropsical fluid is rather poorer in proteids than the plasma, and thus we have no conditions present which would favour the the setting up of diffusion currents. As a matter of fact, we may say that diffusion currents bring about an interchange of constituents between the two fluids, but can under no circumstances effect a total absorption of fluid. We have therefore to inquire whether, under any circumstances, an absorption of dropsical fluids or of isotonic salt solutions can be carried out by the blood vessels. Starling then cites various experiments which show that absorption, as well as transudation through the capillary wall, is determined by the intracapillary pressure. When the pressure rises transudation is increased, when the pressure falls absorption is increased. We have seen that the former may be explained as a simple mechanical filtration, and the question now arises as to the mechanism of the absorption process. Is the absorption effected by the active intervention of the endothelial cells, or are there physical factors at work which will serve to explain it? From the work of Ludwig and Noll we know that the pressure in the lymphatics is extremely low; but, as Starling has shown, there may be considerable pressure in the tissue spaces without communicating itself to the fluid-filling lymphatics. Landerer places the pressure in these spaces at from one-half to three-quarters of the capillary pressure, and then it is evident that if this were the case, and the capillary pressure suddenly sank 50 per cent., the pressure in the extra-vascular spaces would be higher than that in the capillaries, and a backward filtration of lymph might occur. But the fact that the veins are surrounded by the lymph spaces serves to explain the fact that increased tension in the lymph spaces above that in the veins, in regions at any rate investigated by Starling on this point, causes a collapse of these veins, a rise of capillary pressure, and a diminished flow of blood through the part. But Starling has been able to show that the osmotic pressure of proteids within the capillaries is considerable, and is an important factor in causing absorption from the tissues around; and whereas capillary pressure determines transudation, the osmotic pressure of the proteids of the serum determines absorption.

Turning to the factors involved in the causation of dropsy, Starling gives the following list: I.—Factors causing increased transudation.—A. Increased intra-capillary pressure: (*a*) Venous obstruction; (*b*) vaso-dilatation; (*c*) plethora. B. Increased permeability of vessel wall: (*a*) Local injury by mechanical irritants, local injury by thermal irritants, and local injury by chemical irritants; (*b*) malnutrition; (*c*) general injury by circulating poisons (?) C. Watery condition of blood (hydræmia). II.—Factors causing diminished absorption.—A. By lymphatics; (*a*) Paralysis of limbs; (*b*) obstruction of lymphatic trunks. B. By veins: (*a*) Venous obstruction; (*b*) watery condition of blood; (*c*) concentrated transudations. The production of dropsy is almost always due to the concurrent action of two or more of these factors.

The form of dropsy which is simplest in its pathology is that which is due to venous obstruction. Simple venous obstruction, however, will not give rise to œdema unless it be a widespread obstruction involving, say, nearly all the veins of a limb. But venous obstruction when the capillary endothelium has been rendered more permeable by malnutrition or other causes, or when the blood is rendered hydræmic and therefore more readily exuded, is followed by œdema; thus Cohnheim has shown that if a dog be first rendered hydræmic by bleeding repeatedly ligature of the femoral vein is followed by œdema, while ligature of the vein in a normal dog does not give œdema. We may sum up facts bearing on the question by saying that œdema can never be brought about in the limbs by a moderate rise of venous pressure, provided that the capillaries retain their normal permeability. Œdema will occur as soon as the permeability of the vessels is increased. The injury leading to this increase in permeability may be brought about in any of the following ways: (1) Long and continued venous congestion (asphyxia of cells); (2) an excessive rise of intra-capillary pressure breaking down the normal resistance of the cells; and (3) malnutrition due to an impoverished condition of the blood. The production of dropsy in heart disease is next shown to be by no means so simple as it at first appears, but it is due to a complicated series of interacting mechanisms, all of which tend to the death of the organism. We may sum up the sequence of events which ensue on failure of compensation as follows: Stage I.: Heart-pump failure, fall of arterial pressure, rise of pressure in the venous trunks near the heart, fall of capillary pressures in the peripheral parts of the body, in the kidneys, and in the intestines, and absorption of fluid by blood vessels from intestines and peripheral tissues. Stage II.: Continued absorption from the alimentary canal, with diminished excretion from the kidneys, and production of hydræmic plethora, with rise of mean systemic pressure. This leads to Stage III.: Rise of capillary pressure in all dependent parts of the body, capillaries injured by malnutrition, and excessive transudation, leading to dropsy. Stage IV.: The continued hydræmic plethora leads to ever-increasing overfilling of the heart cavities, and to ultimate failure of the already incompetent heart.

We know very little more about the form of dropsy which occurs in renal disease than was known in Cohnheim's time. The discovery of lymphagogues naturally suggests that the change in the vascular endothelium in Bright's disease, which leads to its increased permeability, may be due to the circulation in the blood of some poisonous substance belonging to this group of bodies. This possibility is favoured by the results of one experiment, in which Starling obtained marked quickening of the lymph flow from the thoracic duct after injecting blood serum from a uræmic patient.

Turning back to the Baillie lectures for 1895 on Dropsy we find that Dickinson points out one very significant fact bearing on the question of renal dropsy, viz., that the dropsy liquids, whether of cardiac, renal, or hepatic organs, or that following on the injection of saline fluids, contain between ·783 and ·875 parts per cent. of mineral salts, that is to say that the amount of mineral salts is practically uniform for all these forms of dropsy, but we note that the higher percentage, higher by at most one-tenth per cent., is found in the renal and hepatic forms. On the other hand the percentage of albumen is higher in cardiac than renal dropsy fluid; that it should be highest of all in hepatic dropsy is explained by Starling on anatomical grounds. Now it will be readily conceived that the defective condition of the kidneys may be held to explain the slight increase in the saline constituents in renal dropsy fluid without pointing to any difference in the physics of its production by transudation. How, then, do we account for the lessened percentage of albumen in renal dropsy, seeing that Dickinson regards pressure as the factor *par excellence* which determines the passage of albumen through the membranous walls of the capillaries?

We have already referred to Starling's observations on the consequences of heart pump failure, resulting in hydrœmic plethora and raising of the mean systemic blood pressure above its normal height, so that with the evergrowing balance in favour of absorption of fluid into the blood vessels, the rise of venous pressure extends further and further towards the periphery until the rise also affects the capillary pressure, and in all dependent parts of the body exudation predominates over absorption. Then it would appear that cardiac failure involves increased capillary pressure, combined with hydrœmia, and we see how the lymph effusion will be especially rich in albumen from the secondary effects of malnutrition of the capillary walls.

**Coagulation of the Blood.**—L. Rogers[3] has used the method of Professor Wright for estimating the coagulability of the blood, which consists in pricking a finger and allowing the blood to run into several capillary tubes, and then noting how soon it coagulates so that it cannot be blown out. In normal individuals it coagulates in from two and a half to five minutes, varying slightly with the temperature and the time of day, and especially in relation to meals, being shorter after a full meal and longer after a fast. He conducted a series of experiments which showed that calcium chloride, the inhalation of carbonic acid gas diluted with air by a Kipp's apparatus, increased the coagulability of the blood; thus, with calcium chloride in 15 gr. doses the coagulation of his own blood was reduced from 5¼ to 1¾ minutes. He was further able to show that gallic acid, ergotine (except in the uterine form of hæmorrhage), and potassium iodide have no effect. As regards hæmophilia, it is remarked that the lack of calcium chloride in the blood is probably a very important pathological feature of the disease, and reference is made to the use of this agent and of carbonic acid gas in this disease. The author suggests the use of the vegetable acids or oxygen in cases of threatened or actual thrombosis after typhoid fever, and in other morbid states attended with thromboses.

[1] Brit. Med. Journ., Mar. 21, 1896. [2] Lancet, May 9, 16, and 23, 1896. [3] Lancet, Jan. 4, 1896.

